# Impact of Early Fluid Balance on Long-Term Mortality in Critically Ill Surgical Patients: A Retrospective Cohort Study in Central Taiwan

**DOI:** 10.3390/jcm10214873

**Published:** 2021-10-22

**Authors:** Chieh-Liang Wu, Kai-Chih Pai, Li-Ting Wong, Min-Shian Wang, Wen-Cheng Chao

**Affiliations:** 1 Department of Critical Care Medicine, Taichung Veterans General Hospital, Taichung 40705, Taiwan; cljeff.wu@gmail.com; 2 Department of Computer Science, Tunghai University, Taichung 407224, Taiwan; 3 School of Medicine, Chung Hsing University, Taichung 40227, Taiwan; 4 Department of Industrial Engineering and Enterprise Information, Tunghai University, Taichung 407224, Taiwan; 5 Department of Automatic Control Engineering, Feng Chia University, Taichung 407802, Taiwan; 6 Artificial Intelligence Studio, Taichung Veterans General Hospital, Taichung 40705, Taiwan; minnshyan@vghtc.gov.tw; 7 College of Engineering, Tunghai University, Taichung 407224, Taiwan; kcpai@go.thu.edu.tw; 8 Department of Medical Research, Taichung Veterans General Hospital, Taichung 40705, Taiwan; ting249@vghtc.gov.tw; 9 Big Data Center, Chung Hsing University, Taichung 40227, Taiwan

**Keywords:** critical illness, surgical, fluid balance, mortality, long-term outcome, shock

## Abstract

Fluid balance is an essential issue in critical care; however, the impact of early fluid balance on the long-term mortality in critically ill surgical patients remains unknown. This study aimed to address the impact of day 1–3 and day 4–7 fluid balance on the long-term mortality in critically ill surgical patients. We enrolled patients who were admitted to surgical intensive care units (ICUs) during 2015–2019 at a tertiary hospital in central Taiwan and retrieved date-of-death from the Taiwanese nationwide death registration profile. We used a Log-rank test and a multivariable Cox proportional hazards regression model to determine the independent mortality impact of early fluid balance. A total of 6978 patients were included for analyses (mean age: 60.9 ± 15.9 years; 63.9% of them were men). In-hospital mortality, 90-day mortality, 1-year and overall mortality was 10.3%, 15.8%, 23.8% and 31.7%, respectively. In a multivariable Cox proportional hazard regression model adjusted for relevant covariates, we found that positive cumulative day 4–7 fluid balance was independently associated with long-term mortality (aHR 1.083, 95% CI 1.062–1.105), and a similar trend was found on day 1–3 fluid balance, although to a lesser extent (aHR 1.027, 95% CI 1.011–1.043). In conclusion, the fluid balance in the first week of ICU stay, particularly day 4–7 fluid balance, may affect the long-term outcome in critically ill surgical patients.

## 1. Introduction

Fluid balance is increasingly recognised as an essential issue in critically ill patients [1,2]; however, few studies address the long-term mortality impact of early fluid balance, and data in critically ill surgical patients are particularly sparse [3]. The advance of critical care has led to a steady decrease in hospital mortality among patients admitted to the intensive care unit (ICU) in the past two decades [4], but increasing evidence has shown that patients who survived from critical illness may have sequelae, and die within months after ICU-discharge; therefore, the long-term mortality remains high [5,6,7].

Conservative initial fluid resuscitation and fluid balance is currently an essential issue in fluid management in patients with sepsis [1,8], and one recent study further pointed out the crucial role of fluid balance after reversal of shock, mainly days 4–7 after admission to the ICU, among patients with septic shock [9]. In critically surgical patients, a number of studies focusing on early fluid balance also identified that conservative acute fluid resuscitation and peri-operative fluid management may affect the short-term outcome [3,10,11]. The enhanced recovery after surgery (ERAS) programme is proposed to improve the outcome of surgical patients; however, an optimal peri-operative fluid strategy in critically ill surgical patients remains uncertain in ERAS due to distinct surgical risks and the complex patient group [11,12,13]. Therefore, the optimal fluid strategy after the acute resuscitation and long-term impact on mortality of early fluid balance remains unclear. In the present study, we used two databases, the critical care database of Taichung Veterans General Hospital (TCVGH) and the death registration data of the National Health Insurance Research Database (NHIRD) in Taiwan, to specifically address the day 1–3 and day 4–7 fluid balance status and to investigate the long-term mortality impact of early fluid balance in critically ill surgical patients.

## 2. Materials and Methods

### 2.1. Ethics Approval

Given that the data in the present study were anonymised prior to analyses, the informed consent was waived. This study was approved by the Institutional Review Board of the Taichung Veterans General Hospital (TCVGH: SE20249B) and conducted in accordance with the Declaration of Helsinki.

### 2.2. Study Population

This retrospective cohort study was conducted at TCVGH, a tertiary care referral hospital with 1530 beds and three surgical ICUs in central Taiwan. All adult patients who had been admitted to the ICU between 2015 and 2019 were included in this study, and we used the first ICU admission as the index of ICU admission.

### 2.3. Data Sources and Covariates

We used two databases in the present study: the critical care database of TCVGH and the death registration data of the Taiwanese National Health Insurance Research Database (NHIRD) [14]. Data with regards to demographic information, Acute Physiology and Chronic Health Evaluation (APACHE) II score, surgical departments of ICU stay, ICU admission, daily fluid input and output, medications, mechanical ventilation usage (equal of longer than 3 days), renal replacement therapy, discharge diagnoses and ICU/hospital length of stay were obtained from the TCVGH critical care database. The presence of shock was defined as the use of a vasopressor for more than one day. Data on the date-of-death was retrieved from the death registration data of Taiwanese NHIRD [14]. Given that National Health Insurance is a single-payer and mandatory program with 99.93% coverage of the Taiwanese population in 2019, the date-of-death in the present study is assumed accurate [14,15]. 

### 2.4. Fluid Status

The parameters in this study focusing on fluid status were daily fluid input, output and balance. Input fluid consisted of all intravenous fluid, the volume of drugs dissolved in solution, enteral fluid, parenteral fluids and transfusion of blood products. Output fluid was composed of urine output, haemodialysis and output from drains, orogastric and nasogastric tubes. We presented daily fluid status as daily and cumulative fluid input, output and balance in accordance with the format in previous studies, including our recently published studies [1,16,17,18].

### 2.5. Statistical Analyses

Continuous variables were presented as mean ± standard deviation, and data for categorical variables were shown as frequencies (percentages). The differences between the survivor and non-survivor groups were analysed by the Student’s *t*-test for continuous variables as well as the Chi-square test for categorical variables. Kaplan-Meier analysis was applied to determine the association between mortality and day 1–3/4–7 cumulative fluid balance status. The Cox proportional hazards regression model was applied to identify independent predictors for long-term mortality, and the data were represented by the adjusted hazard ratio (adjHR) and the corresponding 95% confidence interval (CI). Statistical analyses were two-sided, and the level of significance was set at 0.05. Data analyses were conducted using R version 3.6.0.

## 3. Results

### 3.1. Characteristics of the Enrolled Critically Ill Surgical Patients

**Figure 1:**
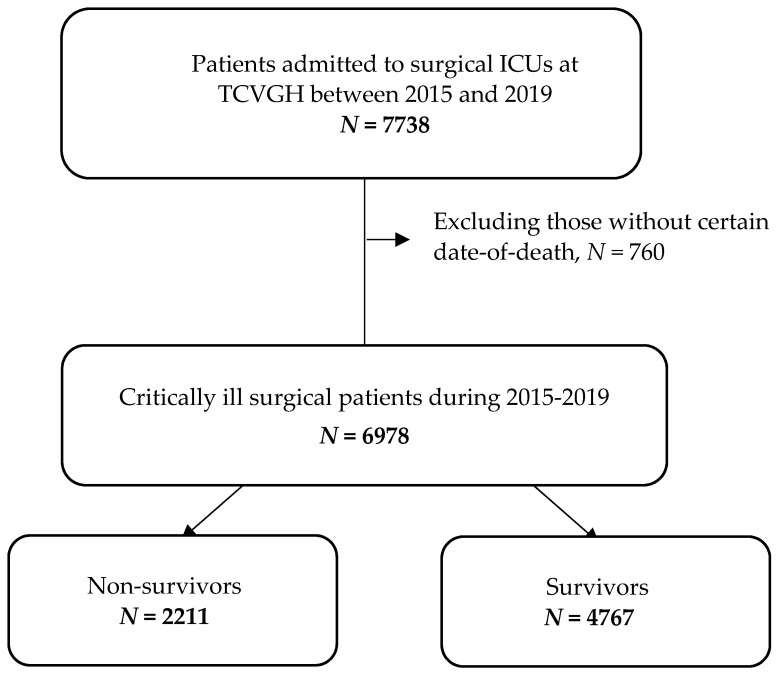
Flow diagram of the enrollment of subjects eligible for analyses.

A total of 6978 patients were eligible for analyses in the present study. The mean age was 60.9 ± 15.9 years, and 63.9% of enrolled patients were male (Figure 1 and Table 1). The main surgical departments of ICU stay included neurosurgery (49.6%), followed by cardiovascular surgery (20.4%), general surgery (6.8%), chest surgery (6.8%) and colon-rectal surgery (5.5%). Compared with survivors, non-survivors tended to have a higher age (66.4 ± 15.3 vs. 58.4 ± 15.5, *p* < 0.01), Charlson comorbidity index (2.3 ± 1.4 vs. 1.3 ± 1.2, *p* < 0.01), APACHE II score (23.2 ± 6.6 vs. 19.0 ± 5.9, *p* < 0.01) and a lower body mass index (BMI) (23.8 ± 4.5 vs. 25.0 ± 4.5, *p* < 0.01). Non-survivors were also more likely to have shock (40.8% vs. 24.0%, *p* < 0.01), to receive mechanical ventilation for more than three days (48.9% vs. 25.7%, *p* < 0.01) and to receive renal replacement therapy during ICU stay (11.6% vs. 2.0%, *p* < 0.01) or end-stage renal disease (1.5% vs. 0.7%), whereas non-survivors were less likely to undergo a surgery during ICU stay (62.0% vs. 76.0%, *p* < 0.01) (Table 1). 

### 3.2. Daily and Cumulative Fluid Status between Day 1 and Day 7

Table 2 shows the first weeks daily and cumulative input (I), output (O) and fluid balance (I-O) data in critically ill surgical patients categorised by overall survival.

The fluid balance appeared to be generally positive on day one (1027.0 ± 1876.7 mL) as well as day two (119.3 ± 1040.8 mL) and then to be balanced or slightly negative on day three (−8.1 ± 923.5 mL), day four (−8.6 ± 827.9 mL) and the following days in the first week of ICU admission. Notably, we found a steady negative fluid balance between day four and day seven in the survivor group, whereas a slightly positive fluid balance was noted in the non-survivor group between days 4–7. Therefore, we divided fluid balance in the first week into day 1–3 fluid balance and day 4–7 fluid balance. Compared with the survivor group, a higher positive cumulative day 1–3 fluid balance was found in the non-survivor group (1607.0 ± 3326.5 vs. 920.8 ± 2266.1, *p* < 0.01) and a similar trend was found with regards to cumulative day 4–7 fluid balance (269.5 ± 2300.3 vs. −145.4 ± 1523.2, *p* < 0.01).

### 3.3. Both Day 1–3 and Day 4–7 Cumulative Fluid Balance Was Correlated with Mortality

**Figure 2:**
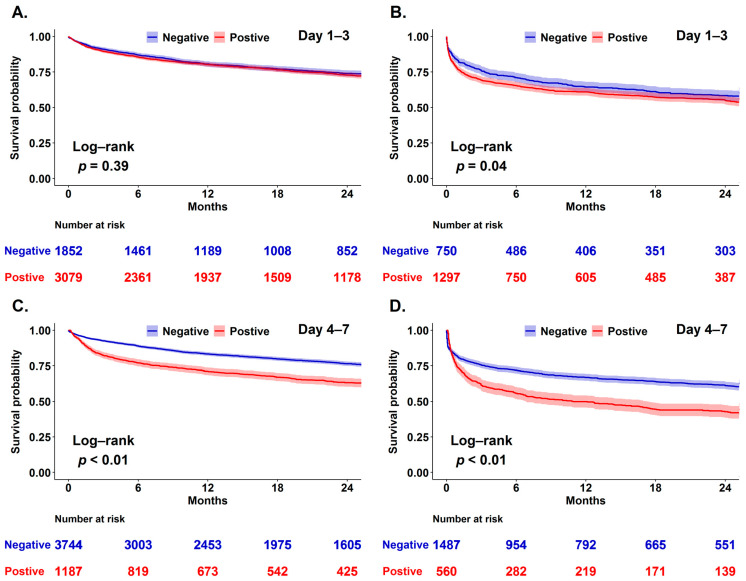
Impact of day 1–3 (**A**,**B**) and day 4–7 (**C**,**D**) fluid balance on long-term mortality among 6978 surgically ill patients with and without shock (Shock: (**A**,**C**); non-shock: (**B**,**D**)).

**Figure 3:**
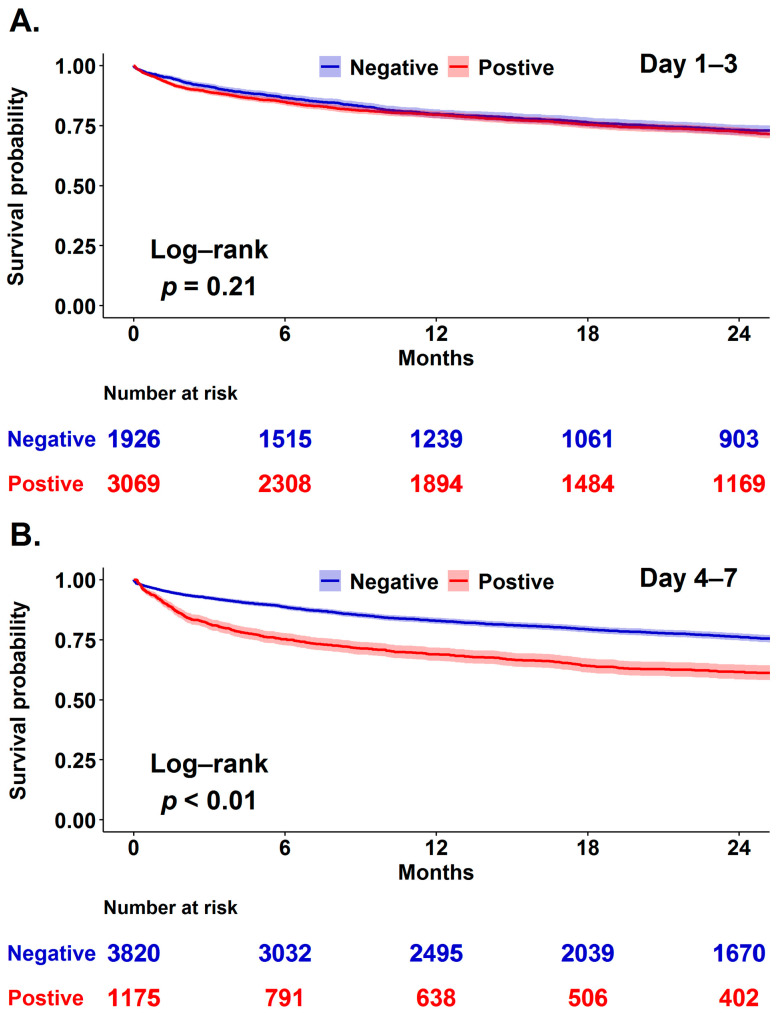
Impact of day 1–3 (**A**) and day 4–7 (**B**) fluid balance on long-term mortality in 4995 critically ill patients receiving surgery.

We then used Kaplan-Meier analysis to explore the correlation between day 1–3 as well as day 4–7 cumulative fluid balance and two-year mortality in critically ill surgical patients categorised by the presence of shock given that shock status may confound the correlation between fluid balance and mortality (Figure 2). We found that day 1–3 cumulative fluid balance tended to be associated with mortality in the patient with shock, but not among those without shock, whereas day 4–7 cumulative fluid balance had a robust mortality impact in patients with and without shock. Given that patients may be admitted to surgical ICUs for underlying diseases or peri/post major surgery critical care, we hence addressed the distinct impacts of day 1–3 and day 4–7 fluid balance on mortality among those receiving surgery during ICU stay (Figure 3). We found that day 4–7 fluid balance, but not day 1–3 fluid balance, was associated with the long-term mortality in patients admitted to ICU for peri/post-operation care. In a multivariate Cox proportional hazard regression model adjusted for age, sex, body mass index, APACHE II score, presence of shock, receiving surgery during ICU stay and relevant covariates, we found that positive cumulative day 4–7 fluid balance (aHR 1.083, 95% CI 1.062–1.105) was independently associated with mortality and a similar trend was found in day 1–3 fluid balance to a lesser extent (aHR 1.027, 95% CI 1.011–1.043) (Table 3). Given that critically ill surgical patients may be admitted due to distinct surgical conditions, we have further conducted subgroup analyses among patients admitted for neurosurgery, cardiovascular surgery and major abdominal surgery (Supplemental Tables S1–S3 and Figures S1–S3). We found that the associations between day 4–7 fluid balance and long-term mortality, the main claim in the present study, were consistent among these three distinct patient groups.

## 4. Discussion

In the present study, we linked the critical care database at TCVGH and death registration data of Taiwanese NHIRD to address the long-term mortality impact of fluid balance in the first week among critically ill surgical patients. We found that not only day 1–3 but also day 4–7 fluid balance was independently associated with long-term mortality. Moreover, the magnitude of the association between day 4–7 fluid balance and mortality appeared to be higher than the mortality association of day 1–3 fluid balance, particularly among those who were admitted for peri/post major surgery. These data highlight the critical role of early fluid balance and the crucial need for a practical protocol to achieve fluid balance in the first week among critically surgically ill patients, particularly those receiving surgery.

The long-term outcome is increasingly recognised as an essential issue in critically ill patients [5,6]. Shankar-Hari et al., analysing a total of 43 studies to investigate the 1-year mortality in critically ill patients who survived from ICU discharge, found that the 1-year post-acute mortality was approximately 16% [19]. The aforementioned data were largely consistent with the finding in this study that one-year post-acute mortality was 15.1% (944/6259). A number of factors have been found to affect the long-term outcome in critically ill surgical patients [20,21]. In the present study, we have adjusted known mortality relevant risk factors, including age, BMI, Charlson comorbidity index, APACHE II score, presence of shock, use of mechanical ventilation as well as receiving renal replacement therapy to demonstrate the independent association of early fluid balance and long-term mortality in critically ill surgical patients. Furthermore, the main finding was consistent in subgroup analyses (Supplemental Tables S1–S3 and Figures S1–S4). 

Indeed, early fluid resuscitation is essential to optimise organ perfusion, but a persistent positive fluid balance has been found to be deleterious in critically ill patients [1,8]. In line with the findings of this study, Acheampong A and Vicent JL found that the persistent positive fluid balance within day-7 of ICU admission was associated with high hospital mortality among 173 critically ill septic patients [1]. Notably, the Sepsis Occurrence in Acutely Ill Patients (SOAP) study investigated 3147 critically ill patients at 198 ICUs in 24 European countries and found that cumulative day 1–3 fluid balance correlated with 60-day mortality [22]. Therefore, there is a crucial need to explore early fluid balance beyond day-3 on the outcome, particularly long-term outcome, in critically ill patients. In the present study, we specifically explored the distinct impact of day 1–3 and day 4–7 fluid balance status on the long-term outcome in 6978 critically ill surgical patients. We found that day 1–3 was slightly associated with mortality in the critically ill surgical patient, particularly those with shock, and day 4–7 had a consistent and robust mortality impact on the critically ill surgical patient with and without shock (Figure 2). These findings highlight the crucial need to explore fluid balance after the stabilisation of critical illness. Similar to our data regarding day 4–7 fluid status, Mourik et al. recently analysed the fluid balance after the reversal of shock among 636 patients with septic shock in the Molecular Diagnosis and Risk Stratification of Sepsis (MARS) project and reported that a 10 mL/kg increase in cumulative fluid balance on ICU-discharge appeared to be associated with a high 30-day (odds ratio (OR), 2.09; 95% CI, 1.64–2.67) and 1-year mortality (OR, 1.53; 95% CI, 1.17–2.01) [9]. In the study of Mourik et al., shock reversal was defined by the discontinuation of vasopressor with a normal serum lactate level, and approximately two days were needed for shock reversal [9]. Therefore, day 4–7 fluid balance should be an appropriate indicator to address the association between post-acute fluid balance and long-term mortality in critically ill patients. Notably, the fining regarding the crucial role of day 4–7 fluid balance is of particular importance given that day 4–7 fluid balance should be a practically modifiable factor to improve long-term outcomes in critically ill surgical patients. Collectively, these findings may support the implementation of restrictive or deresuscitative fluid strategy after initial resuscitation in critically ill surgical patients, although more studies are warranted for optimal measures and parameters targeting fluid removal [23,24].

Morgan recently reported that the increased long-term mortality among critically ill surgical patients might be attributed to multisystem pathophysiological alterations, including impaired respiratory and gastrointestinal function [21]. The fluid overload increases the respiratory workload and may further lead to critical illness-acquired diaphragm dysfunction, which may have a prolonged impact on the respiratory function in critically ill surgical patients [25]. Similarly, the prolonged tissue oedema in the gastrointestinal system may result in diarrhoea, altered microbiota and delayed establishment of nutrient status in critically ill surgical patients [26,27].

Enhanced recovery after surgery (ERAS) programme consists of the patient-centred, evidence-based, multidisciplinary team-developed pathways aiming to improve the outcome of surgical patients; however, an optimal peri-operative fluid strategy in critically ill surgical patients remains uncertain due to distinct surgical risks, varied hemodynamic monitors and complex patient-groups [11,12,13]. Intriguingly, the ERAS pathway has recommended a restrictive fluid approach with a zero peri-operative fluid balance [12]; however, the data of the Restrictive versus Liberal Fluid Therapy in Major Abdominal Surgery (RELIEF) trial showed the vulnerability for acute kidney injury among patients who were unable to receive adequate oral intake and a moderately positive fluid balance of 1–2 litres at the end of surgery might be feasible in patients receiving from major surgery [3,28]. Messin et al. conducted a meta-analysis with 21 randomised control trials and 2729 patients to explore the impact of peri-operative goal-directed therapy aiming to optimise peri-operative fluid management based on the individual patients’ hemodynamic response and found that peri-operative goal-directed therapy slightly reduced the incident rate of post-operative complications (−0.10; 95% CI −0.14–−0.007), but not mortality, mainly short-term hospital mortality [11]. The aforementioned, at least partly, inconclusive evidence reflect that early fluid is somehow complex in critically ill surgical patients with distinct risk and highlight the need for addressing the impact of post-acute (day 4–7 in this study) fluid balance on the long-term mortality in critically ill surgical patients receiving surgery. In the present study, we specifically demonstrated the crucial role of day 4–7 fluid balance and suggest that day 4–7 fluid balance be considered a part of the EARS protocol.

Given that the majority of the enrolled patients in this study were critically ill neurological patients, we hence further investigated the long-term mortality impact of early fluid balance in these patients. We validated a consistent trend that both day 1–3 and day 4–7 were independently associated with long-term mortality in critically ill neurosurgical patients, and the magnitude appeared to be slightly lower than those in the other surgical patients (Supplemental Figure S1 and Table S1). We postulate that the aforementioned finding may result from neurocritical patients being less likely to have indications for a large volume of fluid resuscitation. Similar to the evolving concept of conservative early fluid resuscitation and fluid balance in sepsis [1,29], the fluid strategy changes in critically ill neurological patients [30]. For example, euvolemia is currently recommended in patients with brain injury, given increasing evidence found that both hypovolemia and hypervolemia are associated with unfavourable outcomes [30]. Vergouw et al. investigated 246 patients with aneurysmal subarachnoid haemorrhage and reported that an increased early day 1–3 fluid input was associated with the incidence of delayed cerebral ischemia [31]. Similarly, Rass et al. recently reported that a higher day 1–2 fluid input, instead of day 1–2 fluid balance, was associated with the outcome, including prolonged mechanical ventilation, early brain oedema, anaemia, delayed cerebral ischemia and 3-month functional status, in 237 patients with non-traumatic subarachnoid haemorrhage enrolled between 2010 and 2016. Notably, in line with our data, Rass et al. illustrated daily fluid balance from day-1 to day-15 after the incident of subarachnoid haemorrhage and explicitly demonstrated that fluid balance generally reaches a stable status on approximately day-6/day-7 after the subarachnoid haemorrhage, suggesting the crucial role of fluid balance in critically ill neurological patients [32]. Unlike increasing studies that have shown a slightly increased comorbidity and mortality rate among critically ill neurological patients with a positive fluid balance in days 1–3, evidence of the impact of fluid balance beyond day-3 is sparse [31,33]. Hence, the data in the present study provide crucial evidence regarding the long-term mortality impact of day 4–7 fluid balance in critically ill surgical patients.

There are limitations to our study. First, given the observational nature of the present study, the decision of fluid and shock management was made by the individual attending physician and could potentially introduce a confounding effect. However, given the administration of intensivists in the reported ICUs, the aforementioned concern should at least partly be mitigated. Second, being a single-centre observational study, external validation is hence needed. However, the coding of fluid status and relevant data is in accordance with our previous published studies regarding fluid status in critically ill influenza and cancer patients [16,18]. Third, the presence of shock was defined by using vasopressors and could not further delineate subtypes of shock, such as cardiogenic or septic shock. Fourth, there should be unmeasured potential confounding factors, such as diuretics and the duration of vasopressors. 

## 5. Conclusions

In conclusion, we linked databases at TCVGH and Taiwanese NHIRD to investigate the impact of early fluid balance on long-term mortality in critically ill surgical patients. We found that days 1–3 slightly affected the long-term mortality in critically ill surgical patients with shock. Notably, we identified that day 4–7 fluid balance had a consistent impact on the long-term mortality in critically ill patients with and without shock, and the aforementioned impact was robust among those receiving surgery. These findings highlight the essential role of early fluid balance in critically ill surgical patients and support the implementation of restrictive or deresuscitative fluid strategy, particularly on days 4–7 after the stabilisation of the initial critical illness. More studies are warranted to prospectively confirm our findings and to explore the underlying mechanism.

## Figures and Tables

**Table 1:** Characteristics of enrolled critically ill surgical patients categorised by overall mortality.

	All	No Survival	Survival	*p*-Value
	(*N* = 6978)	(*N* = 2211)	(*N* = 4767)	
Basic characteristics				
Age (years)	60.9 ± 15.9	66.39 ± 15.3	58.39 ± 15.54	<0.01
Sex (male)	4459 (63.9%)	1530 (69.2%)	2929 (61.4%)	<0.01
Body mass index	24.6 ± 4.5	23.8 ± 4.5	25 ± 4.5	<0.01
Charlson comorbidity index	1.6 ± 1.3	2.3 ± 1.4	1.3 ± 1.2	<0.01
Surgical departments				<0.01
Neurosurgery	3463 (49.6%)	845 (38.2%)	2618 (54.9%)	
Cardiovascular surgery	1421 (20.4%)	263 (11.9%)	1158 (24.3%)	
General surgery	474 (6.8%)	226 (10.2%)	248 (5.2%)	
Chest surgery	476 (6.8%)	235 (10.6%)	241 (5.1%)	
Colorectal surgery	386 (5.5%)	241 (10.9%)	145 (3.0%)	
Otorhinolaryngology surgery	206 (3%)	132 (6.0%)	74 (1.6%)	
Urology	173 (2.5%)	109 (4.9%)	64 (1.3%)	
Plastic surgery	97 (1.4%)	45 (2.0%)	52 (1.1%)	
Others	282 (4%)	115 (5.2%)	167 (3.5%)	
Severity and managements				
APACHE II score	20.3 ± 6.4	23.2 ± 6.6	19.0 ± 5.9	<0.01
Presence of shock	2047 (29.3%)	902 (40.8%)	1145 (24.0%)	<0.01
Use of mechanical ventilation	2307 (33.1%)	1081 (48.9%)	1226 (25.7%)	<0.01
Receiving surgery during admission	4995 (71.6%)	1371 (62.0%)	3624 (76.0%)	<0.01
Emergency surgery	1019 (14.6%)	334 (15.1%)	685 (14.4%)	0.42
Renal replacement therapy (RRT)				
Temporal RRT during admission	353 (5.1%)	257 (11.6%)	96 (2.0%)	<0.01
RRT for ESRD	67 (1%)	32 (1.5%)	35 (0.7%)	<0.01
Outcomes				
ICU-stay, days	8.7 ± 11.3	12.14 ± 15.34	7.07 ± 8.38	<0.01
Hospital-stay, days	22.4 ± 24.2	29.37 ± 33.08	19.1 ± 17.71	<0.01
Ventilator-day	7.87 ± 12.13	12.3 ± 15.4	5.5 ± 9.1	<0.01
Mortality at distinct time points				<0.01
In-hospital mortality	719 (10.3%)	719 (32.5%)	NA	NA
90-day mortality	1103 (15.8%)	1103 (49.9%)	NA	NA
1-year mortality	1663 (23.8%)	1663 (75.2%)	NA	NA
Follow-up period, years	1.7 ± 1.4	0.7 ± 0.91	2.17 ± 1.34	<0.01

Abbreviations: APACHE II, acute physiology and chronic health evaluation; RRT, renal replacement therapy; ESRD, end-stage renal disease; ICU, intensive care unit.

**Table 2:** Daily and cumulative 1–3 and 4–7 fluid status of the 6978 critically ill surgical patients categorised by mortality.

	All (N = 6978)	Non-Survivors (N = 2211)	Survivors (N = 4767)	*p*-Value
Daily fluid balance (mL)				
Day 1	1027.0 ± 1876.7	1254.7 ± 2285.5	921.4 ± 1642.7	<0.01
Day 2	119.3 ± 1040.8	235.7 ± 1226.8	65.3 ± 937.4	<0.01
Day 3	−8.1 ± 923.5	116.6 ± 1058.6	−66.0 ± 847.5	<0.01
Day 4	−8.6 ± 827.9	89.8 ± 974.5	−54.2 ± 746.0	<0.01
Day 5	−3.0 ± 750.0	77.6 ± 915.5	−40.3 ± 656.1	<0.01
Day 6	−4.0 ± 679.0	52.3 ± 859	−30.0 ± 575.0	<0.01
Day 7	1.6 ± 627.6	49.8 ± 790.3	−20.8 ± 534.3	<0.01
Cumulative fluid balance (mL)				
Day 1–3	1138.2 ± 2667.4	1607.0 ± 3326.5	920.8 ± 2266.1	<0.01
Day 4–7	−13.9 ± 1817.8	269.5 ± 2300.3	145.4 ± 1526.2	<0.01

**Table 3:** Cox proportional hazard regression analysis for mortality.

Characteristics	Univariable		Multivariable	
	HR (95% CI)	*p*-Value	HR (95% CI)	*p*-Value
Age, per 1 year increment	1.029 (1.026–1.032)	<0.001	1.010 (1.007–1.013)	<0.001
Sex (male)	1.303 (1.190–1.426)	<0.001	1.238 (1.130–1.356)	<0.001
BMI, per 1 year increment	0.946 (0.937–0.956)	<0.001	0.945 (0.936–0.955)	<0.001
CCI, per 1 year increment	1.469 (1.433–1.505)	<0.001	1.298 (1.263–1.335)	<0.001
APACHE II score, per 1 year increment	1.116 (1.108–1.124)	<0.001	1.068 (1.060–1.077)	<0.001
Presence of shock	2.005 (1.842–2.183)	<0.001	1.597 (1.460–1.746)	<0.001
Use of mechanical ventilation	2.306 (2.121–2.507)	<0.001	1.260 (1.148–1.384)	<0.001
Surgery during ICU admission	0.541 (0.497–0.590)	<0.001	0.604 (0.553–0.660)	<0.001
Temporal RRT during ICU admission	4.398 (3.858–5.015)	<0.001	1.874 (1.625–2.162)	<0.001
RRT for ESRD	1.808 (1.275–2.563)	0.001	0.953 (0.670–1.357)	0.789
Cumulative day 1–3 fluid balance *	1.104 (1.088–1.121)	<0.001	1.027 (1.011–1.043)	0.001
Cumulative day 4–7 fluid balance *	1.139 (1.113–1.165)	<0.001	1.083 (1.062–1.105)	<0.001

* Per 1 litre increment. Abbreviations: HR: hazard ratio; CI: confidence interval; BMI, body mass index; CCI, Charlson comorbidity index; APACHE II, acute physiology and chronic health evaluation II; ICU, intensive care unit; RRT, renal replacement therapy; ESRD, end-stage renal disease.

## Data Availability

The data in the present study are available upon request from the corresponding author.

## Supplementary Materials

The following are available online at https://www.mdpi.com/article/10.3390/jcm10214873/s1, Figure S1. Association of fluid balance with long-term outcome among 3463 neurocritical patients. Figure S2. Association of fluid balance with long-term outcome among 1421 patients admitted for cardiovascular surgery. Figure S3. Association of fluid balance with long-term outcome among 860 patients admitted for major abdominal surgery. Figure S4. Impact of fluid balance on long-term mortality in critically ill surgical patient with and without renal replacement therapy. Table S1. Cox proportional hazard regression analysis for mortality in 3463 neurocritical patients. Table S2. Cox proportional hazard regression analysis of mortality among 1421 patients admitted for cardiovascular surgery. Table S3. Cox proportional hazard regression analysis of mortality among 860 patients admitted for major abdominal surgery.
