# Supplementary material for: Impact of Early Fluid Balance on Long-Term Mortality in Critically Ill Surgical Patients: A Retrospective Cohort Study in Central Taiwan
Source: J Clin Med. 2021 Oct 22;10(21):4873. doi: 10.3390/jcm10214873 (PMC8584411; doi:10.3390/jcm10214873)
Supplement: Supplementary file 1 [file jcm-10-04873-s001.zip › jcm-1408784-supplementary.pdf]

**Supplemental Table S1. Cox proportional hazard regression analysis for mortality in 3,463 neurocritical patients**

| Characteristics | Univariable | | Multivariable | |
|---|---|---|---|---|
| | **HR (95% CI)** | ***P* value** | **HR (95% CI)** | ***P* value** |
| Age, per 1 year increment | 1.035 (1.031-1.040) | <0.001 | 1.004 (1.000-1.008) | 0.050 |
| Sex (male) | 1.280 (1.113-1.472) | 0.001 | 1.173 (1.040-1.324) | 0.009 |
| BMI, per 1 year increment | 0.945 (0.929-0.962) | <0.001 | 0.936 (0.924-0.948) | <0.001 |
| CCI, per 1 year increment | 1.461 (1.398-1.526) | <0.001 | 1.311 (1.266-1.358) | <0.001 |
| APACHE II score, per 1 year increment | 1.140 (1.124-1.155) | <0.001 | 1.060 (1.050-1.070) | <0.001 |
| Presence of shock | 2.396 (2.057-2.790) | <0.001 | 1.273 (1.139-1.424) | <0.001 |
| Use of mechanical ventilation | 2.278 (1.990-2.607) | <0.001 | 1.201 (1.063-1.357) | 0.003 |
| Surgery during ICU admission | 0.570 (0.497-0.654) | <0.001 | 0.572 (0.510-0.643) | <0.001 |
| Temporal RRT during ICU admission | 5.014 (3.732-6.738) | <0.001 | 1.900 (1.615-2.235) | <0.001 |
| RRT for ESRD | 2.798 (1.394-5.614) | 0.004 | 0.843 (0.560-1.269) | 0.412 |
| Cumulative day 1-3 fluid balance* | 1.122 (1.087-1.158) | <0.001 | 1.034 (1.016-1.052) | <0.001 |
| Cumulative day 4-7 fluid balance* | 1.137 (1.085-1.191) | <0.001 | 1.103 (1.078-1.128) | <0.001 |

*Per 1 litre increment. Abbreviations: HR: hazard ratio; CI: confidence interval; BMI, body mass index; CCI, Charlson comorbidity index; APACHE II, acute physiology and chronic health evaluation II; ICU, intensive care unit; RRT, renal replacement therapy; ESRD, end-stage renal disease.

**Supplemental Table S2. Cox proportional hazard regression analysis of mortality among 1,421 patients admitted for cardiovascular surgery**

| Characteristics | Univariable | | Multivariable | |
|---|---|---|---|---|
| | HR (95% CI) | *P* value | HR (95% CI) | *P* value |
| Age, per 1 year increment | 1.058 (1.046-1.070) | <0.001 | 1.041 (1.029-1.054) | <0.001 |
| Sex (male) | 0.990 (0.763-1.285) | 0.940 | 1.107 (0.846-1.449) | 0.459 |
| BMI, per 1 year increment | 0.949 (0.920-0.978) | 0.001 | 0.946 (0.916-0.977) | 0.001 |
| CCI, per 1 year increment | 1.568 (1.461-1.683) | <0.001 | 1.279 (1.178-1.390) | <0.001 |
| APACHE II score, per 1 year increment | 1.116 (1.093-1.140) | <0.001 | 1.042 (1.019-1.065) | <0.001 |
| Presence of shock | 1.453 (1.140-1.851) | 0.003 | 0.990 (0.757-1.295) | 0.944 |
| Use of mechanical ventilation | 3.661 (2.848-4.707) | <0.001 | 2.172 (1.619-2.914) | <0.001 |
| Temporal RRT during ICU admission | 6.962 (5.107-9.492) | <0.001 | 3.606 (2.518-5.165) | <0.001 |
| RRT for ESRD | 1.381 (0.570-3.346) | 0.475 | 0.705 (0.284-1.749) | 0.451 |
| Cumulative day 1-3 fluid balance* | 1.136 (1.084-1.192) | <0.001 | 1.026 (0.980-1.074) | 0.277 |
| Cumulative day 4-7 fluid balance* | 1.232 (1.114-1.363) | <0.001 | 1.119 (1.039-1.205) | 0.003 |

*Per 1 litre increment. Abbreviations: HR: hazard ratio; CI: confidence interval; BMI, body mass index; CCI, Charlson comorbidity index; APACHE II, acute physiology and chronic health evaluation II; ICU, intensive care unit; RRT, renal replacement therapy; ESRD, end-stage renal disease.

**Supplemental Table S3. Cox proportional hazard regression analysis of mortality among 860 patients admitted for major abdominal surgery**

| Characteristics | Univariable | | Multivariable | |
|---|---|---|---|---|
| | HR (95% CI) | *P* value | HR (95% CI) | *P* value |
| Age, per 1 year increment | 1.018 (1.013-1.024) | <0.001 | 1.006 (0.999-1.013) | 0.080 |
| Sex (male) | 1.279 (1.053-1.555) | 0.013 | 1.268 (1.040-1.545) | 0.019 |
| BMI, per 1 year increment | 0.970 (0.952-0.989) | 0.002 | 0.950 (0.931-0.970) | <0.001 |
| CCI, per 1 year increment | 1.322 (1.255-1.393) | <0.001 | 1.269 (1.200-1.343) | <0.001 |
| APACHE II score, per 1 year increment | 1.070 (1.054-1.085) | <0.001 | 1.048 (1.031-1.065) | <0.001 |
| Presence of shock | 1.717 (1.430-2.062) | <0.001 | 1.412 (1.157-1.723) | 0.001 |
| Use of mechanical ventilation | 1.128 (0.902-1.411) | 0.291 | 0.812 (0.644-1.023) | 0.078 |
| Temporal RRT during ICU admission | 2.573 (2.006-3.302) | <0.001 | 1.712 (1.310-2.239) | <0.001 |
| RRT for ESRD | 1.333 (0.689-2.579) | 0.393 | 1.167 (0.599-2.275) | 0.651 |
| Cumulative day 1-3 fluid balance* | 1.076 (1.050-1.102) | <0.001 | 1.053 (1.025-1.082) | <0.001 |
| Cumulative day 4-7 fluid balance* | 1.119 (1.084-1.156) | <0.001 | 1.088 (1.053-1.124) | <0.001 |

*Per 1 litre increment. Abbreviations: HR: hazard ratio; CI: confidence interval; BMI, body mass index; CCI, Charlson comorbidity index; APACHE II, acute physiology and chronic health evaluation II; ICU, intensive care unit; RRT, renal replacement therapy; ESRD, end-stage renal disease.

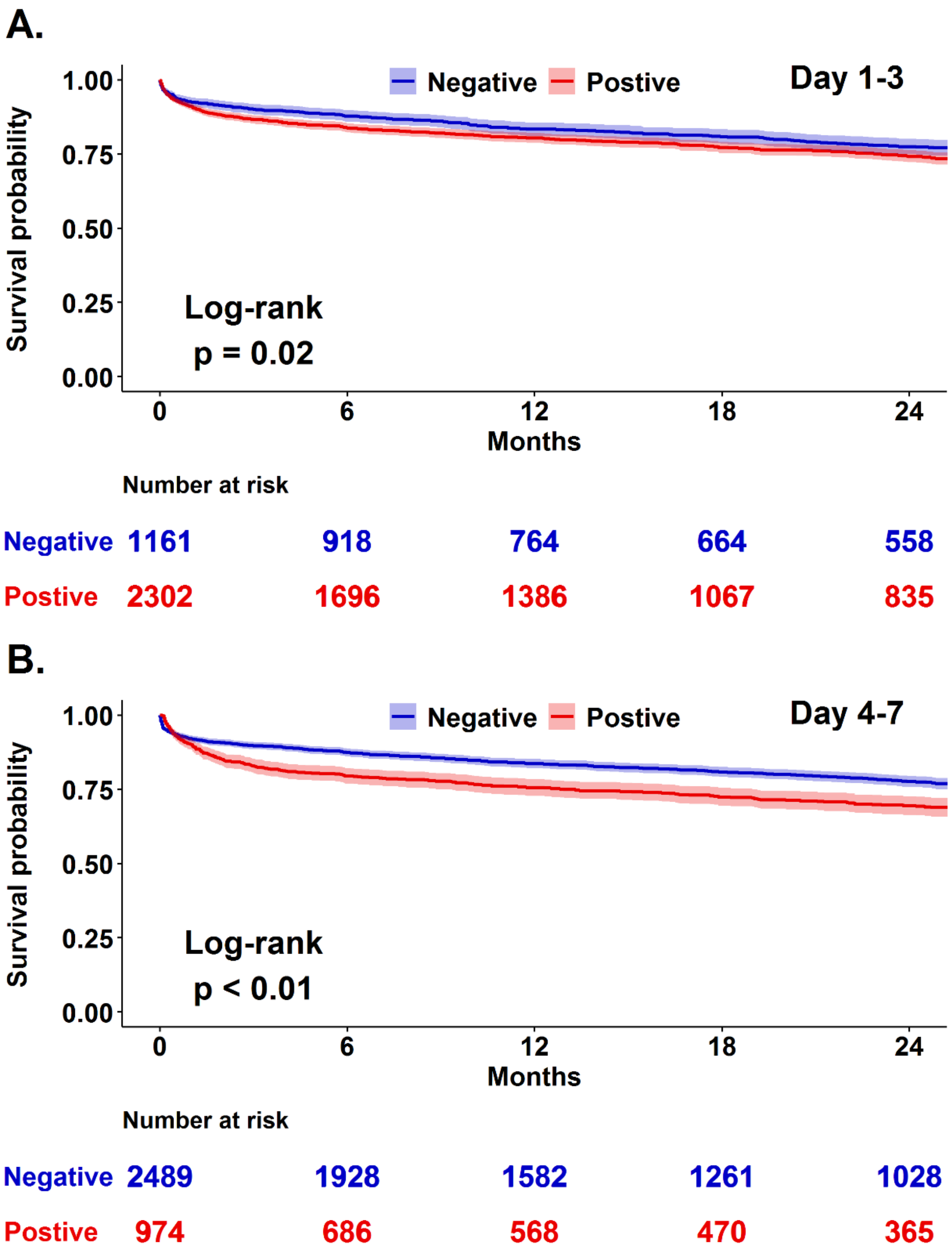


**Supplemental Figure S1.** Association of day 1-3 (A) and day 4-7 (B) fluid balance with long-term outcome among 3,463 neurocritical patients

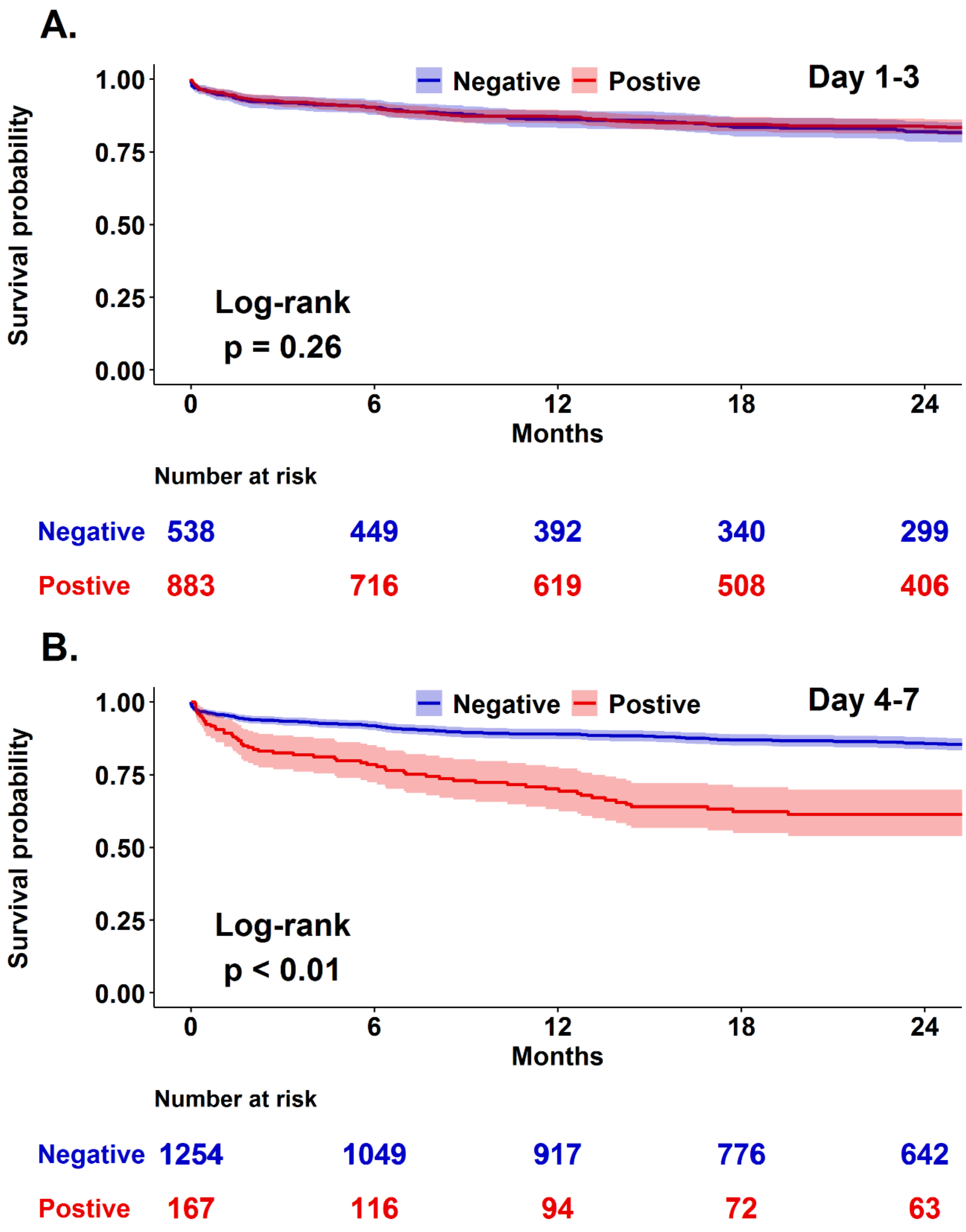


**Supplemental Figure S2. Association of day 1-3 (A) and day 4-7 (B) fluid balance with long-term outcome among 1,421 patients admitted for cardiovascular surgery**

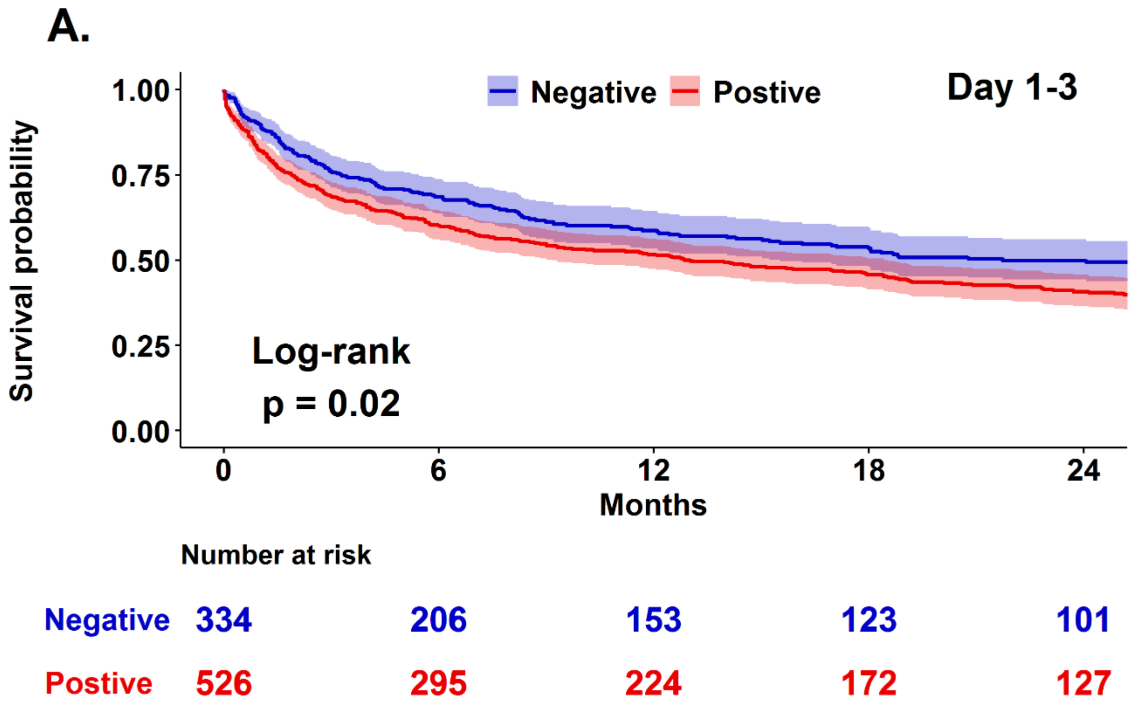


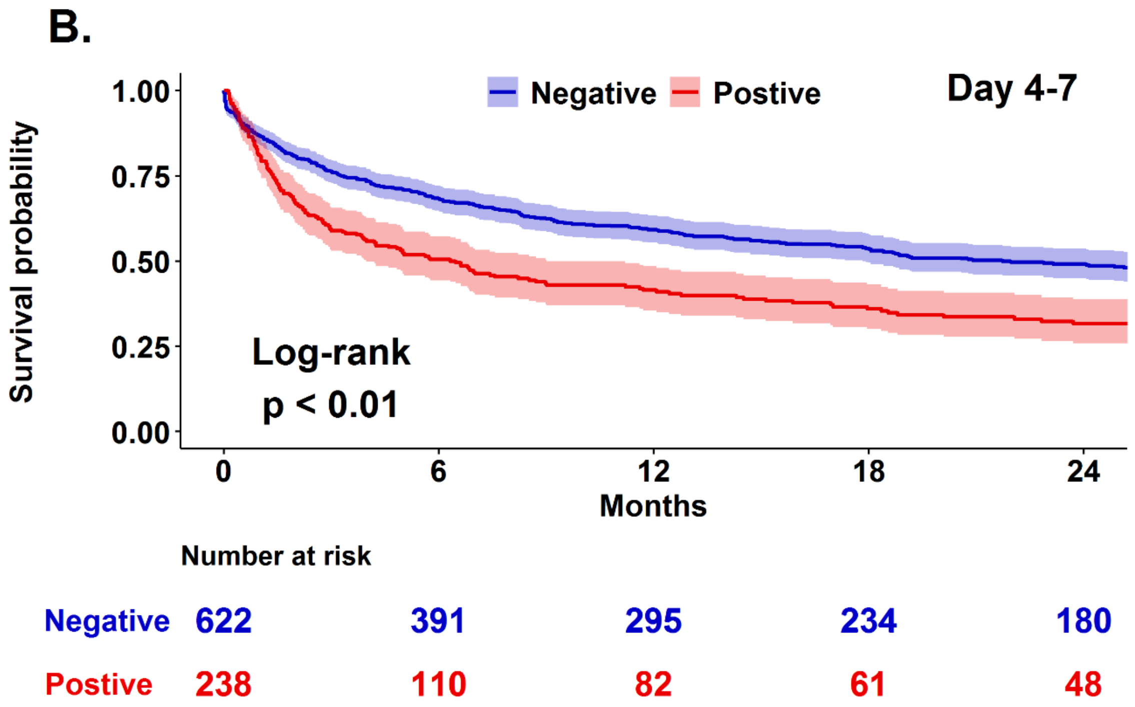


**Supplemental Figure S3. Association of day 1-3 (A) and day 4-7 (B) fluid balance with long-term outcome among 860 patients admitted for major abdominal surgery**

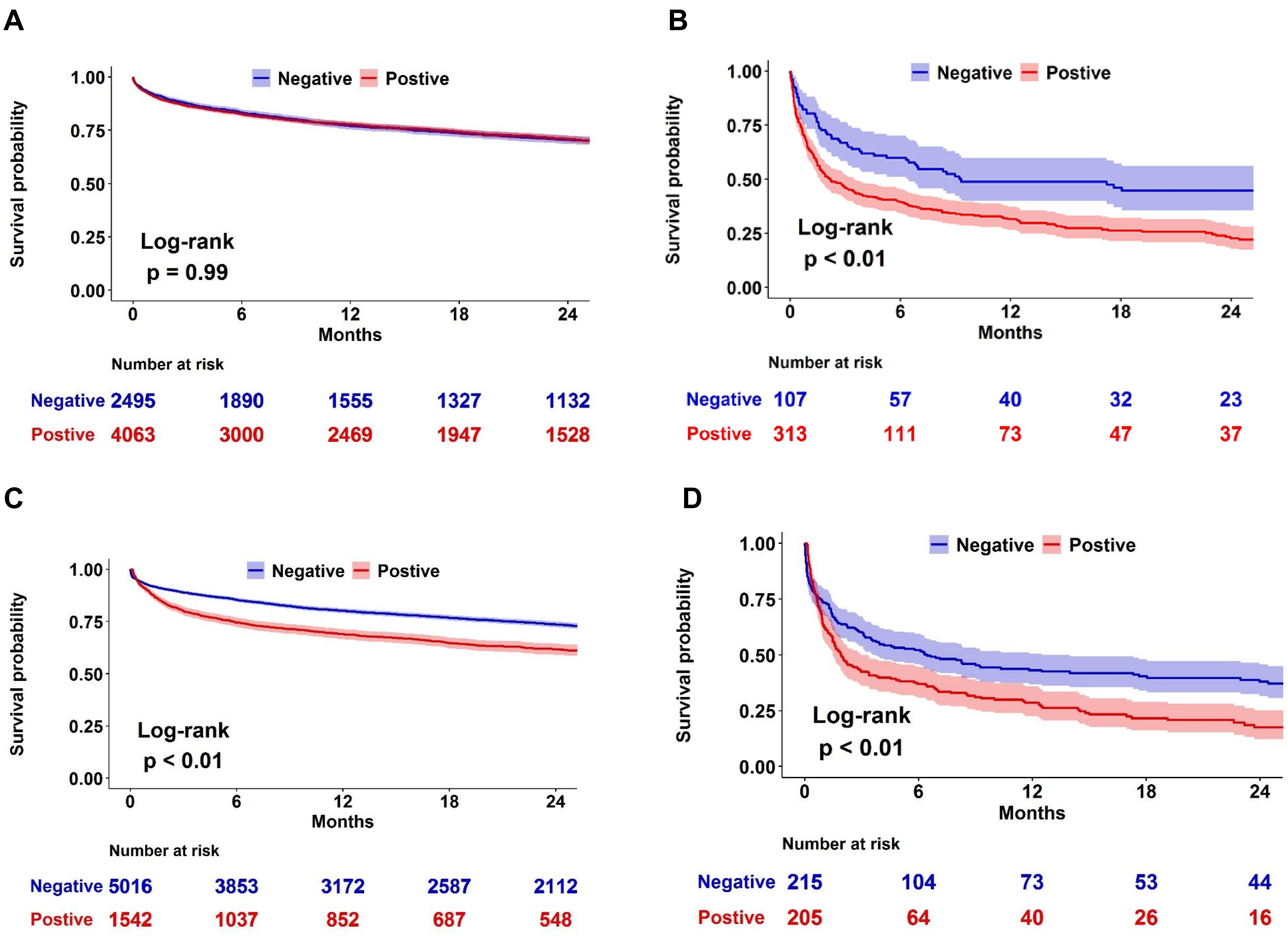


**Supplemental Figure S4. Impact of day 1-3 (A, B) and day 4-7 (C, D) fluid balance on long-term mortality in critically ill surgical patient with and without renal replacement therapy (RRT) (Non-RRT: A, C; RRT: B, D)**
